# Nivolumab-Induced Pneumonitis in a Patient With Urothelial Cancer

**DOI:** 10.7759/cureus.39511

**Published:** 2023-05-26

**Authors:** Arbab Furquan Ud din Kasi, Mohammad Imran Nagi, Burhanuddin A Kasi

**Affiliations:** 1 Internal Medicine, King Edward Medical University, Lahore, PAK; 2 Internal Medicine, Liaquat University of Medical and Health Sciences, Karachi, PAK; 3 Internal Medicine, Dow University of Health Sciences, Karachi, PAK

**Keywords:** transitional cell carinoma, urothelial cell carcinoma, methylprednisolone, eisenhower medical center, case report, programmed death-1 (pd-1) inhibitors, cancer treatment, interstitial pneumonitis, opdivo, nivolumab

## Abstract

The introduction of immune checkpoint inhibitors has revolutionized cancer treatment. These drugs function by inhibiting the binding of programmed death-1 (PD-1) and its ligand, PD-L1, which inhibits the immune response against cancer cells. Nivolumab is a PD-1 inhibitor that specifically targets the PD-1 pathway. The main side effects of these drugs are unpredictable immune-related toxicities that occur when self-reactive T cells are abnormally activated and cause inflammation in various organs. The organs most often affected are the endocrine glands, lungs, skin, and gut. Recognizing and addressing lung inflammation is crucial, particularly in individuals with lung cancer. However, it can be challenging to diagnose due to the distinctive features of their disease and treatment regimen.

This case report presents a 66-year-old man with a medical history of hypertension, chronic kidney disease (stage 3A), hypothyroidism, type 2 diabetes mellitus (DM), and transitional cell carcinoma of the bladder with interstitial pneumonitis secondary to nivolumab. The patient presented to the Eisenhower Medical Center, Rancho Mirage, CA, with dyspnea and cough for two weeks. He received methylprednisolone (Solu-Medrol) at 1.0 mg/kg for immune checkpoint inhibitor-induced pneumonitis and was discharged on 1 liter (L)/min home-oxygen therapy along with prednisone 50 mg twice daily (BD) for six weeks, trimethoprim-sulfamethoxazole (Bactrim) DS BD, and pantoprazole (Protonix) 40 mg once daily. Subsequently, nivolumab therapy was discontinued. At his follow-up visit two weeks later, he felt well and did not need oxygen therapy at rest.

## Introduction

The development of checkpoint inhibitors has transformed the treatment paradigm for cancer. Nivolumab binds programmed death-1 (PD-1) with high affinity, blocks its interactions with programmed cell death-ligand 1 (PD-L1) and programmed cell death-ligand 2 (PD-L2), and stimulates memory response to tumor antigen-specific T-cell proliferation [1]. It is currently Food and Drug Administration (FDA)-approved for the treatment of melanoma, non-small cell lung cancer (NSCLC), renal cell carcinoma (RCC), squamous cell carcinoma (SCC) of the head and neck, pleural mesothelioma, small cell lung cancer (SCLC), urothelial carcinoma, colorectal cancer (CRC) with microsatellite instability-high (MSI-H) or mismatch repair-deficient (dMMR), hepatocellular carcinoma (HCC), classical Hodgkin's lymphoma, and esophageal squamous cell carcinoma (ESCC) [2]. Despite the key role of nivolumab in regulating the immune response, it is associated with serious immune-related adverse events (irAEs). These adverse effects include hypothyroidism, hypopituitarism, adrenal insufficiency, diarrhea, colitis, rash, encephalitis, Guillain-Barré syndrome, and pneumonitis.

According to a meta-analysis, the incidence of pneumonitis associated with using PD-1 inhibitors - specifically nivolumab and pembrolizumab - as a single therapy was 2.7% for any grade and 0.8% for grade ≥3 [3]. The clinical symptoms include fever, cough, chest pain, and dyspnea. The diagnosis is typically supported by CT findings ranging from cryptogenic organizing pneumonia-like, ground-glass opacities, interstitial, and hypersensitivity. The grading of pneumonitis severity is done according to the National Cancer Institute's Common Terminology Criteria for Adverse Events (CTCAE). In this case report, we focus on bringing pneumonitis to the forefront of clinicians' attention by describing the clinical course of the condition and the CT scan image findings observed in a patient who developed it after receiving nivolumab monotherapy.

## Case presentation

A 66-year-old man presented to the emergency department of the Eisenhower Medical Center, Rancho Mirage, CA, with a cough and dyspnea for two weeks. He used to work as a truck driver and a mailman. His medical history included hypertension, bi-fascicular block, chronic kidney disease (stage 3A), hypothyroidism, type 2 diabetes mellitus (DM), and transitional cell carcinoma of the bladder (T1 N0 M0) and renal pelvis (T3 N0 M0). His surgical history was significant for a right nephroureterectomy, which he had undergone nine months ago. His medications included insulin, amlodipine, and levothyroxine. He had received four cycles of cisplatin + gemcitabine; his last cycle had been 10 months ago. In addition, he had been taking nivolumab 480 mg intravenous (IV) once a month for seven months. His last dose of nivolumab had been eight weeks ago. He had a 30-pack-year smoking history, which he had quit 24 years ago, but he did not consume alcohol or use recreational drugs. His family history was insignificant except for hypertension in his mother.

The patient had developed a dry cough about four months ago. Three weeks ago, he had been treated for coronavirus disease 2019 (COVID-19) and bilateral lower lobe pneumonia. He had completed treatment with remdesivir, steroids, and antibiotics and was on room air when discharged. Although he had felt well for the first week after returning home, he soon developed shortness of breath that slowly worsened over the following two weeks. Subsequently, he came to our hospital and was admitted to the medical ward for three days. The patient was alert and oriented but appeared anxious. On reviewing his systems, fatigue and weight loss were observed. The physical examination revealed rales in bilateral middle and lower lung fields. All other examinations were unremarkable. His vital examination showed a temperature of 98.2 °F (36.7 °C), blood pressure of 135/87 mmHg, heart rate of 114/min, and a respiratory rate of 22/min. His oxygen saturation on room air was 82%, which improved to 94% on 6 liters (L)/min of oxygen.

**Figure 1:**
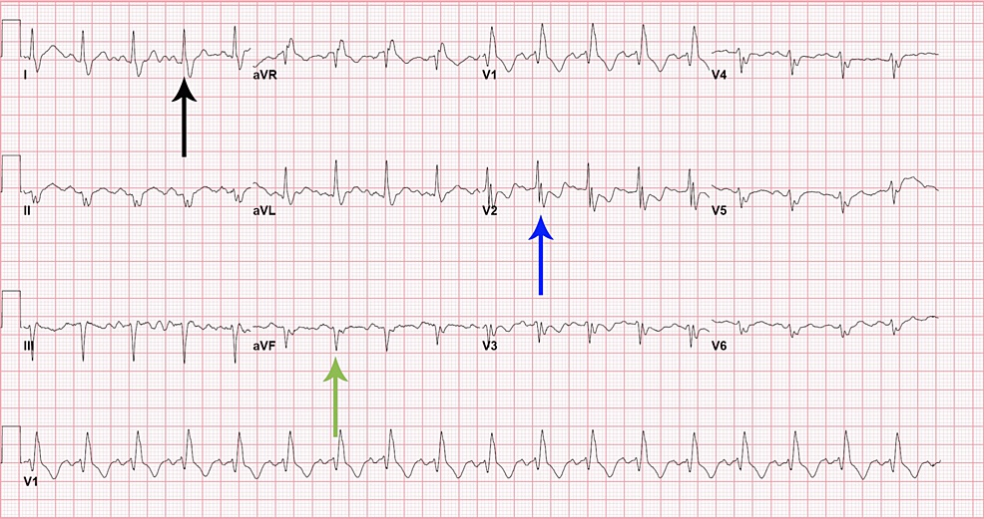
ECG of the patient showing bi-fascicular block Positive QRS in lead I (black arrow) + negative QRS in avF (green arrow) showing left axis deviation. RSR pattern in lead V2 (blue arrow) showing right bundle branch block (RBBB) ECG: electrocardiogram

**Figure 2:**
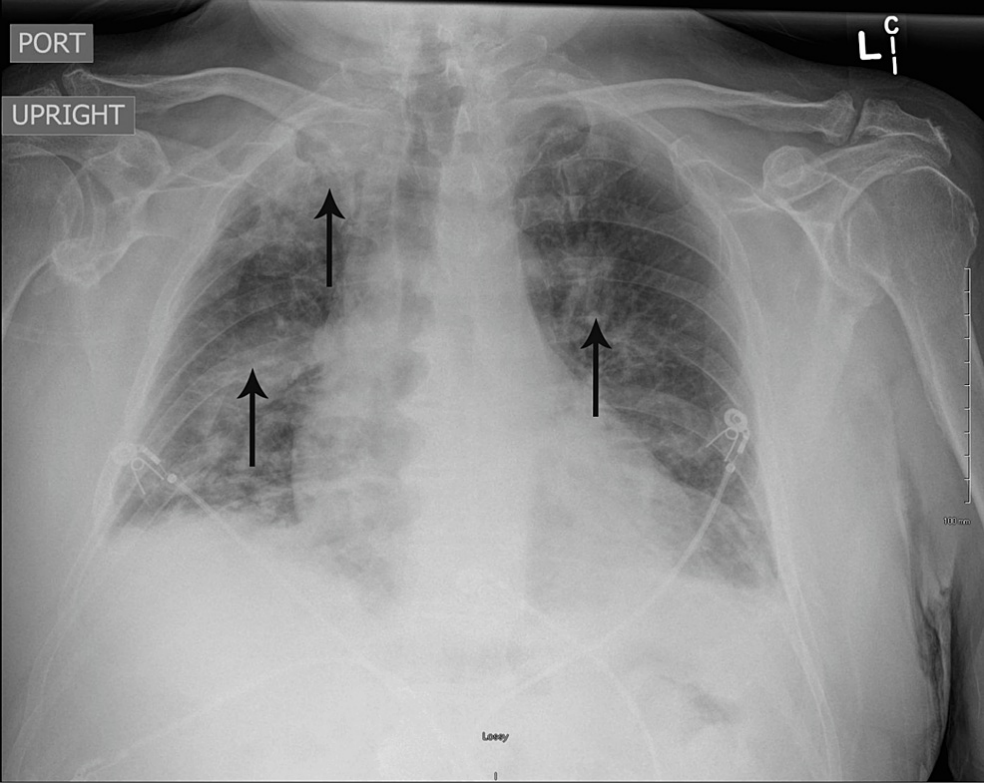
Chest X-ray showing bilateral lung opacities (arrows)

**Figure 3:**
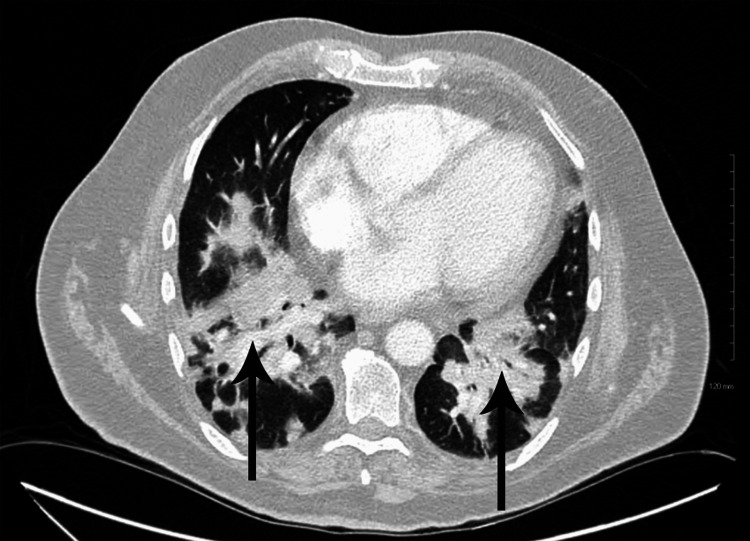
CT scan of the chest with contrast showing bilateral lung opacities (arrows) CT: computed tomography

**Figure 4:**
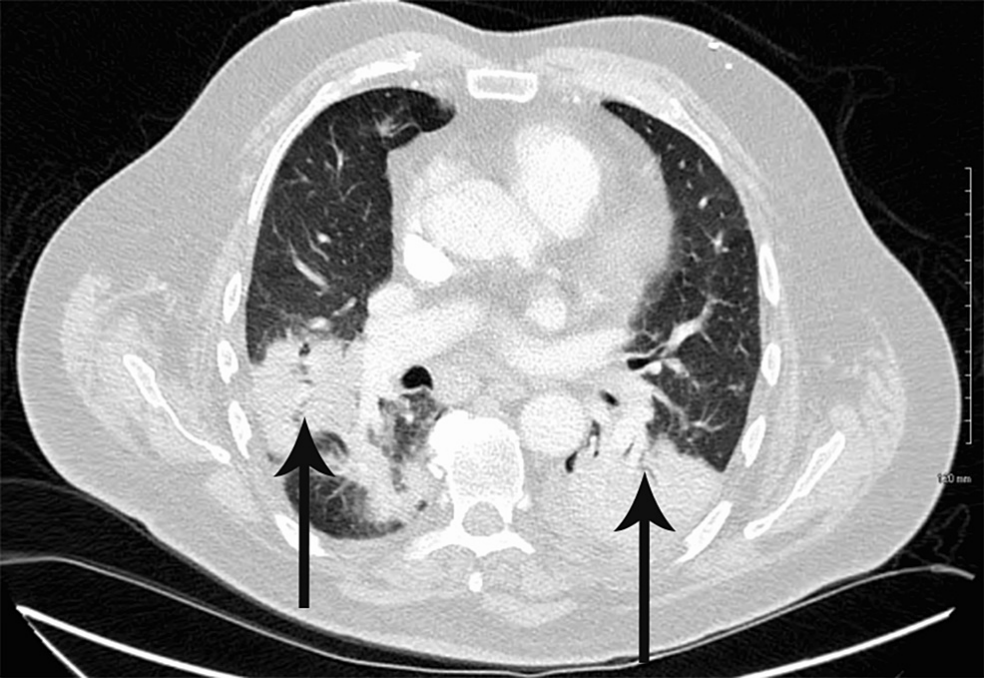
CT scan of the chest with contrast showing bilateral lung opacities (arrows) CT: computed tomography

**Figure 5:**
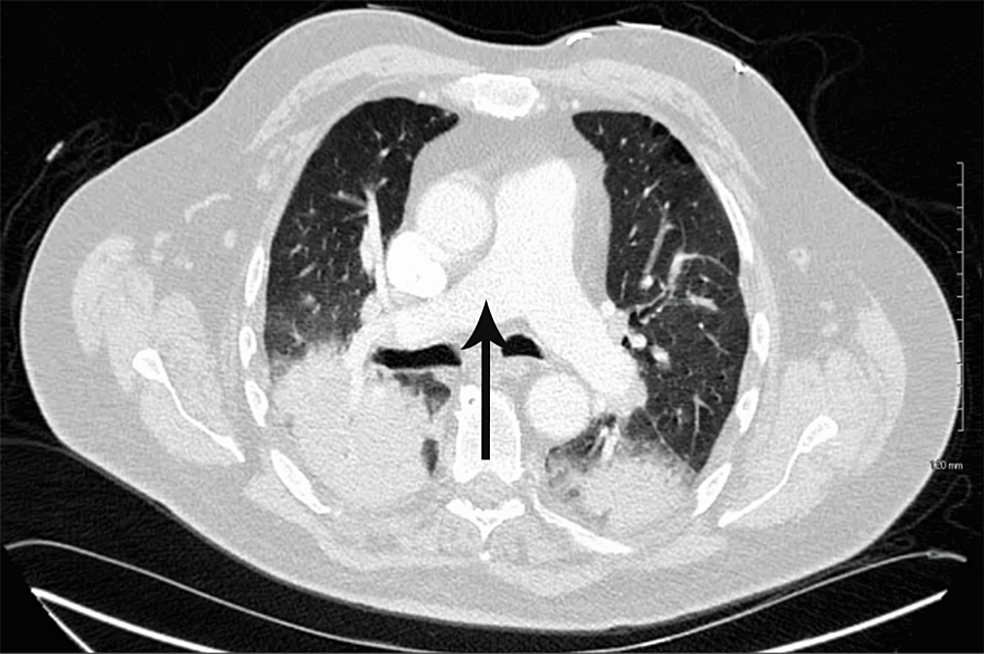
CT scan of the chest with contrast showing no evidence of pulmonary embolism (arrow) CT: computed tomography

The laboratory tests, including complete blood count (CBC), complete metabolic panel (CMP), and blood cultures, were ordered, which were within normal limits. Polymerase chain reaction (PCR) of severe acute respiratory syndrome coronavirus 2 (SARS‑CoV‑2), respiratory syncytial virus (RSV), and influenza A and influenza B were negative. An echocardiogram was performed, which was normal except for a mildly dilated aortic root (4.1 cm). The electrocardiogram (ECG) revealed sinus tachycardia and a bi-fascicular block (Figure 1). An upright chest X-ray was positive for bilaterally heterogenous airspace opacities within the lungs (Figure 2). The chest CT scan showed heterogenous airspace consolidation scattered throughout both lungs (Figures 3, 4). There was no evidence of pulmonary embolism (Figure 5). A pleural fluid culture and analysis were within normal limits. These findings most likely represented a drug-induced lung disease/organizing pneumonia-type pattern as the patient had developed symptoms after starting nivolumab treatment and all other causes of cough and dyspnea were ruled out.

**Figure 6:**
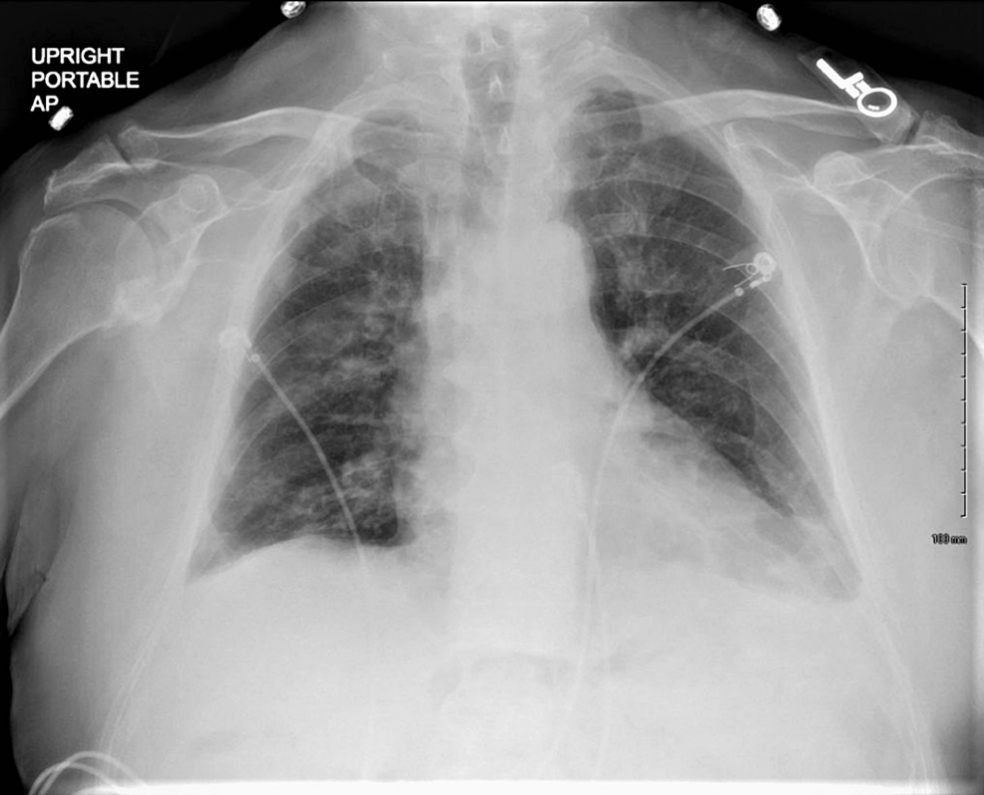
Follow-up chest X-ray showing decreased lung opacities bilaterally

Subsequently, immune checkpoint inhibitor-induced pneumonitis (grade 3) was diagnosed on the basis of the National Cancer Institute's CTCAE. The patient was given Solu-Medrol at 1 mg/kg. His oxygen requirement improved from 6 L/min on admission to 1 L/min at rest and 3 L/min on exertion over the following week. He was discharged on 1 L/min home-oxygen therapy along with prednisone 50 mg twice daily (BD) for six weeks, trimethoprim-sulfamethoxazole (Bactrim) double strength (DS) BD, and pantoprazole (Protonix) 40 mg once daily, with a plan to taper off the steroids afterward. In addition, nivolumab therapy was discontinued. Two weeks later, he was seen in the office and no longer required oxygen therapy at rest. His chest X-ray showed improvement with decreased bilateral lung opacities (Figure 6).

## Discussion

Nivolumab is an immune checkpoint inhibitor that works by targeting PD-1. On December 22, 2014, the FDA granted accelerated approval to nivolumab (OPDIVO; Bristol-Myers Squibb, New York, NY) for the treatment of patients with unresectable or metastatic melanoma and disease progression following ipilimumab and, if BRAF V600 mutation is positive, a BRAF inhibitor [4]. Indications were expanded to encompass other types of solid tumors, including urothelial, hepatocellular, and RCC. Nivolumab-induced pneumonitis is an uncommon but potentially severe toxicity. It typically manifests within a median duration of 2.8 months (range: nine days to 19.2 months) [5]. Clinical symptoms include fever, cough, chest pain, and dyspnea. It presents different variations in imaging. Our patient had bilateral lower lung opacities, which can also be seen in pneumonia. Therefore, clinicians need to consider the possibility of drug-induced pneumonitis in patients receiving nivolumab therapy. Naidoo et al. reported the following five distinct radiologic subtypes in which it can manifest: cryptogenic organizing pneumonia (COP)-like [5/27 (19%)], ground glass opacities [GGO; 10/27 (37%)], interstitial [2/27 (7%)], hypersensitivity [6/27 (22%)], and pneumonitis not otherwise specified [4/27 (15%)] [6].

In summary, screening and monitoring these adverse events regularly before and during nivolumab therapy is recommended. Early detection and treatment of side effects are vital when using nivolumab and other checkpoint inhibitors. The National Cancer Institute's CTCAE is utilized to grade the severity of pneumonitis symptoms to establish consistent terminology for treatment-related adverse events (Table 1).

**Table 1:** General grading guidelines from CTCAE ADL: activities of daily living; CTCAE: Common Terminology Criteria for Adverse Events

CTCAE grade	Description
1	Asymptomatic; clinical or diagnostic observations only; intervention not indicated
2	Symptomatic; medical intervention indicated; limiting instrumental ADL
3	Severe symptoms; limiting self-care ADL; oxygen indicated
4	Life-threatening respiratory compromise: urgent intervention indicated (e.g., tracheotomy or intubation)
5	Death

The clinical practice guideline of the American Society of Clinical Oncology suggests prescribing prednisone at 1-2 mg/kg per day and gradually reducing it by 5-10 mg/kg over four to six weeks for managing grade 2 pneumonitis. For grade 3 pneumonitis, IV administration of methylprednisolone at a rate of 1-2 mg/kg per day, with gradual tapering over four to six weeks, along with empirical antibiotics and discontinuation of the offending agent is recommended [7].

## Conclusions

This case report presented a patient who developed interstitial pneumonitis secondary to nivolumab. He was successfully treated with systemic corticosteroids and discontinuation of nivolumab. Healthcare professionals should be aware of the potential for pneumonitis in patients undergoing treatment with immune checkpoint inhibitors. Early detection and management of immune-related toxicities can improve patient outcomes and quality of life. The aim of this case report is to bring nivolumab-induced pneumonitis to the forefront of clinicians' attention and review the treatment outcomes.
